# Of molecules, memories and migration: M1 acetylcholine receptors facilitate spatial memory formation and recall during migratory navigation

**DOI:** 10.1098/rspb.2018.1904

**Published:** 2018-11-14

**Authors:** Timothy C. Roth, Aaron R. Krochmal

**Affiliations:** 1 Department of Psychology, Franklin and Marshall College, PO Box 3003, Lancaster, PA 17603, USA; 2 Department of Biology, Washington College, 300 Washington Avenue, Chestertown, MD 21620, USA

**Keywords:** learning, memory, critical period, turtle

## Abstract

Many animals use complex cognitive processes, including the formation and recall of memories, for successful navigation. However, the developmental and neurological processes underlying these cognitive aspects of navigation are poorly understood. To address the importance of the formation and recollection of memories during navigation, we pharmacologically manipulated turtles (*Chrysemys picta*) that navigate long distances using precise, complex paths learned during a juvenile critical period. We treated freely navigating turtles both within and outside of their critical learning period with a specific M1 acetylcholine receptor antagonist, a drug known to disrupt spatial cognition. Experienced adult turtles lost all navigational ability under the influence of the drug, while naive juveniles navigated successfully. We retested these same juveniles the following year (after they had passed their critical period). The juveniles that initially navigated successfully under the influence of the antagonist (but were unable to form spatial memories) were unable to do so subsequently. However, the control animals (who had the opportunity to form memories previously) exhibited typical navigational precision. These results suggest that the formation of spatial memories for navigation occur during a critical period, and successful navigation after the critical period is dependent upon the recall of such memories.

## 1. Introduction

Many species use aspects of cognition (herein defined as ‘all processes involved in acquiring, storing, and using information from the environment’ [1]) to navigate complex environments [2]. In migratory species, for example, spatial learning and other cognitive experiences may impact the success in locating alternative seasonal habitats [3–8]. However, the role of cognitive development and the neurological mechanisms that underlie cognitive processing during migration are unclear.

Several studies highlight the importance of experience for learning migratory routes (e.g. [5,7,9]), though the nature and role of these experiences in successful navigation are poorly studied. Some authors suggest a role of spatial memory and learning in the creation of migratory patterns (e.g. [5,7,9]), though it is difficult to dissociate spatial memory from other factors, both cognitive (e.g. non-spatial memories, motivation) and non-cognitive (e.g. sensation, perception, or attention). This difficulty is in part a function of the logistics of experimentally manipulating the neurological and physiological processes that govern cognition.

Recent advances in behavioural neuroscience, stemming particularly from pharmacological manipulation of specific neural receptor types, have greatly expanded our understanding of the cognitive processes and neurochemical mechanisms influencing animal movements (e.g. [10,11]). By targeting specific receptors in the brain, researchers can directly manipulate the neurological and physiological processes involved in the generation and modulation of behaviours while simultaneously observing the complex spatial behaviours that arise. Specifically, numerous studies in a variety of taxa suggest that navigation behaviours are controlled, at least in part, by spatial cognitive processes regulated by the cholinergic system ([12–14]; but see [15]). Pharmacological manipulation with scopolamine, a general muscarinic acetylcholine receptor (AChR) antagonist, successfully blocks the formation and recall of spatial memories during laboratory experiments of navigation [14,16–18]. Moreover, scopolamine alters the precision of animal movements in the field during migration [19,20], supporting the role of the cholinergic system, and potentially spatial cognition, in large-scale migratory movements. Such studies have been critical to our understanding of how the brain produces spatially explicit memories, and how such memories influence navigation behaviours.

Despite this support for the role of ACh in spatial cognition, the complexity of the cholinergic system limits our ability to fully understand the role of spatial cognition in navigation. Given the breadth of muscarinic receptor types (M1–M5), and the confounding impact of simultaneously blocking these receptors (e.g. alterations in thirst, light sensitivity, and motivation) with a generalist antagonist (reviewed by Klinkenberg & Blokland [15]), it is possible that the disruptions in navigation observed during these migratory studies (e.g. [19,20]) could have resulted from the manipulation of non-spatial, non-cognitive processes such as sensation, perception, or attention. Thus, such studies could not definitively conclude that observed changes in navigation were solely due to a manipulation of spatial memory.

Recent evidence suggests a particularly important role of the M1 AChR in facilitating complex, higher-order cognitive processing, such as working memory (e.g. [21,22]). M1 AChRs are associated with regulating postsynaptic excitability of neurons and are abundant in the hippocampus [23], indicating a potential role in spatial cognition, the loss of which has been implicated in age-related cognitive impairment (e.g. [22,24–26]). However, the relevance of M1 AChRs for the processes associated with navigation during migration has not been tested experimentally.

We used our system of semi-aquatic turtles as a model of high-precision navigation to examine the role of spatially explicit memories and the development and recall of such memories as facilitated by M1 AChR. Turtles in this system experience seasonally ephemeral water sources (e.g. [27,28]). After their habitat is lost, resident turtles navigate to alternative aquatic habitats using very precise (±3.5 m), highly predictable routes (animals use the same 300–1200 m routes year after year [27,28]). These paths must be learned during a critical period prior to age 4 [28], after which turtles are unable to learn or follow routes [20,28,29]. We hypothesize that naive juveniles form memories of their navigation routes during the critical period and then use spatial memory recall to navigate as adults. Moreover, we hypothesize that this pattern of spatial learning is facilitated by M1 AChRs.

## 2. Results and discussion

### (a) Adult navigation hinges on M1 AChR-dependent memory recall

In our system, successful navigation in adulthood depends on prior experiences [20,28]. However, how early experiences translate to successful navigation as an adult can be through a variety of mechanisms (e.g. the recall of spatial and non-spatial memories and path imprinting). To investigate the role of memory recall in adult turtle navigation, we monitored freely navigating turtles *via* radiotelemetry using well-established protocols [20,28,29]. While monitoring turtle movements, we performed pharmacological manipulations of spatial memory (July 2016). Because experienced animals follow the same precise route year after year, we allowed turtles to navigate their routes naturally prior to manipulation. Once adult turtles (*n* = 7; four female, three male) navigated approximately 1/4–1/2 of their paths, they were injected with VU0255035 (N-(3-oxo-3-(4-(pyridine-4-yl)piperazin-1-yl)propyl)benzo[c][1,2,5]thiadiazole-4-sulfonamide hydrate; 1.3 mg kg^−1^ in 2% v/v dimethyl sulfoxide (DMSO)/saline; IP; Sigma-Aldrich), a specific M1 muscarinic acetylcholine receptor (M1 AChR) antagonist known to disrupt spatial memory in mammals under laboratory conditions [24,25]. As a control, we injected adults (*n* = 3; one female, two male) with a DMSO vehicle (2% v/v with saline) at identical dosage volumes. If the antagonist disrupts spatial memory, then we predict that adults should deviate from the traditional paths; control animals should navigate the historic paths with typical precision.

**Figure 1.**
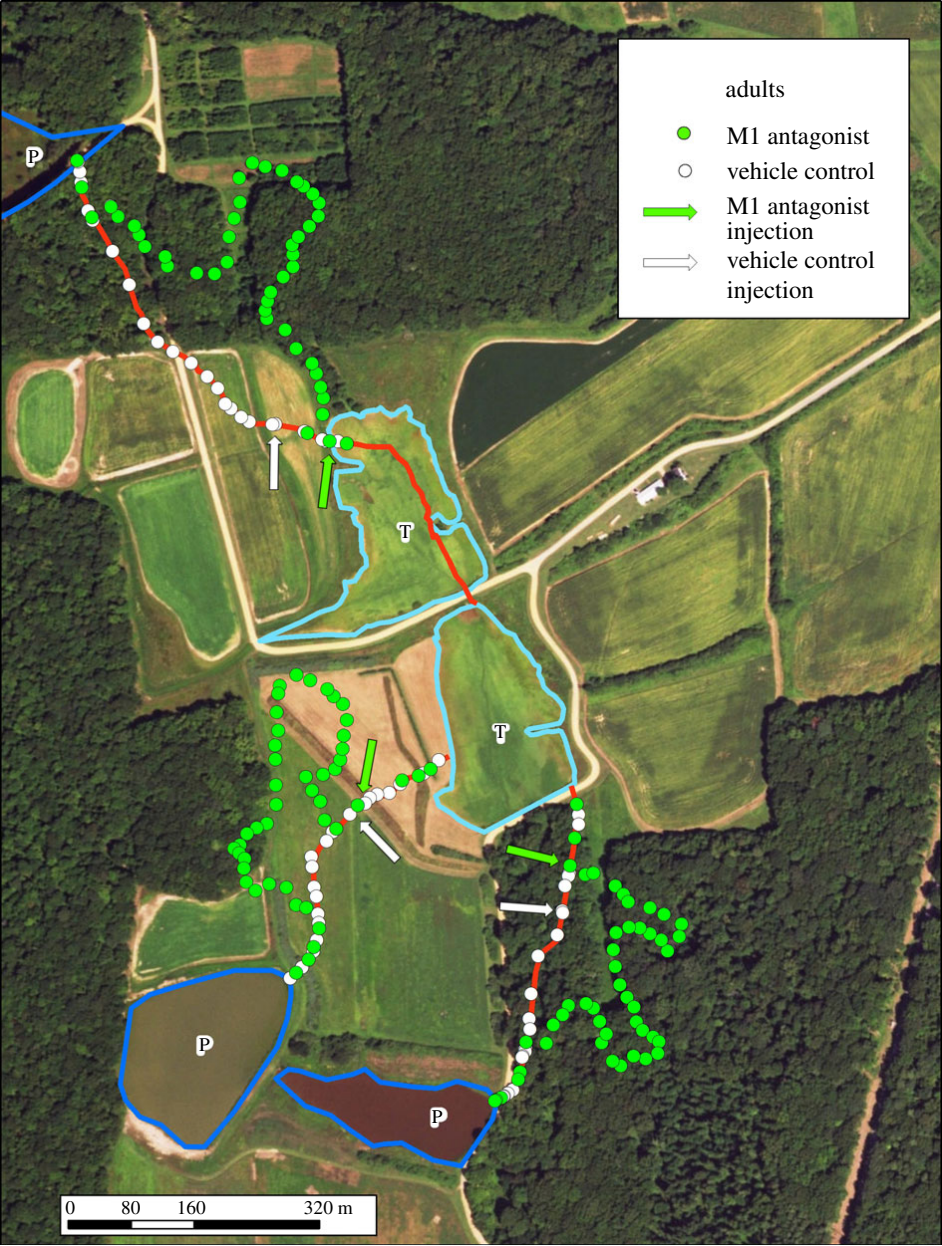
Successful navigation is based on M1 AChR-facilitated memory recall in adult turtles. Representative sample of movement of adult *Chrysemys picta* from temporary (T) to permanent (P) ponds while treated with either an M1 AChR antagonist or DMSO control. All control adults (figure 1, *n* = 3, white) followed traditional, predicted paths (red). Movement of all adults receiving the treatment M1 antagonist (figure 1, *n* = 7, green) drifted dramatically away (greater than 200 m) from the traditional routes. Arrows represent the location of injection. Each line of points represents the movement of one individual. (Online version in colour.)

**Figure 2.**
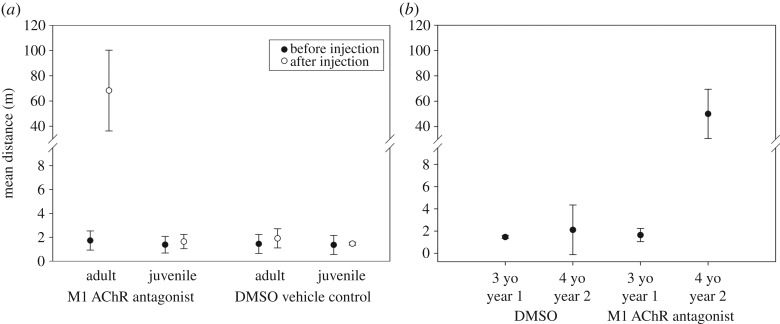
The precision of navigation is a function of cognitive processing in adult turtles. Distance (m) of navigating adult and naive juvenile *Chrysemys picta* from the traditional resident paths [22,23] by treatment. All turtles from all groups maintained high precision of movement prior to injection of the M1 antagonist or DMSO control, with no significant deviation from the traditional paths (*a*). After injection, adults in the M1 antagonist treatment deviated significantly from the traditional paths (*a*). By contrast, control adults and juveniles in the critical period continued to navigate with high precision (*a*,*b*). However, the following year, juveniles that had passed the critical period deviated significantly from traditional paths when treated previously with the antagonist, but not with DMSO (*b*). Points are means ± 95% CI.

Before treatment, all turtles demonstrated high navigational precision on their historical paths (mean overlap with the 3.5 m historic buffer was not different among M1, DMSO, juvenile, or historical groups; *F*_3,12_ = 0.200, *p =* 0.894; figure 1). Upon treatment with the M1 AChR antagonist, adults were disoriented and moved off of their paths, roaming significantly away from their predicted, historical route (mean overlap with the 3.5 m buffer in the M1 treatment group was significantly less than that of the DMSO, juveniles, or historical groups; *F*_3,12_ = 846.105, *p* < 0.001; figures 1 and 2). We note that this response was robust, occurring in all habitat types and at all locations where adults received the M1 AChR antagonist. By contrast, all turtles in the DMSO control group continued to follow the traditional paths, with high accuracy (mean overlap with the 3.5 m historic buffer was not significantly different; *p* > 0.999; figures 1 and 2).

The striking effect of the M1 AChR antagonist was temporary. All but one treated adult regained precise navigational abilities, returned to their paths within 6–8 h, and navigated to their alternative aquatic habitats. A single turtle wandered so far off of its route that it was unable to return to its target pond; being concerned for its welfare (and following IACUC protocol), we retrieved this animal and returned it to the pond at the end of its original route. The following year, this animal returned to the temporary pond on its own, and completed the seasonal migration, demonstrating a lack of negative long-term effects of the drug treatment.

### (b) Naive juvenile navigation is not dependent on M1 AChR

In our system, juveniles (less than 4 years old (yo)) can successfully navigate paths without prior experience on or memory of the paths [20,28]. Prior studies suggest that these naive individuals seem to learn to navigate following a ground-based, local, multi-modal cue for navigation [20,28]. Thus, naive juveniles can be used to control for possible non-spatial cognitive effects of the M1 AChR antagonist on navigation (e.g. sensory reception, motivation, attention, imprinting).

We trapped naive juvenile turtles in a distinct population (hereafter ‘donor population’) and released them at our study site. The two sites are approximately 18.5 km apart, a distance that greatly exceeds even the largest overland movements in *C. picta* (e.g. [30]), and are not connected by water. Thus, we were assured that the navigational ability of these translocated turtles was not influenced by prior experience (i.e. animals were naive [28]).

We pharmacologically manipulated and radiotracked naive juveniles in the same manner as adults. We allowed naive juveniles to begin overland navigation and navigate approximately 1/4–1/2 of their route, at which time we injected them with either the M1 AChR antagonist (*n* = 6; all 3 yo; three female, three male) or DMSO as a vehicle control (*n* = 3; one 2 yo, male; two 3 yo, one female, one male) at the same dosage concentrations used in adults. As naive juveniles lack memory of the paths, yet can still navigate the paths based on local cues [20,28], if the antagonist disrupts navigation-relevant behaviours or processes other than memory, then we predict that naive juveniles should deviate from the traditional paths.

**Figure 3.**
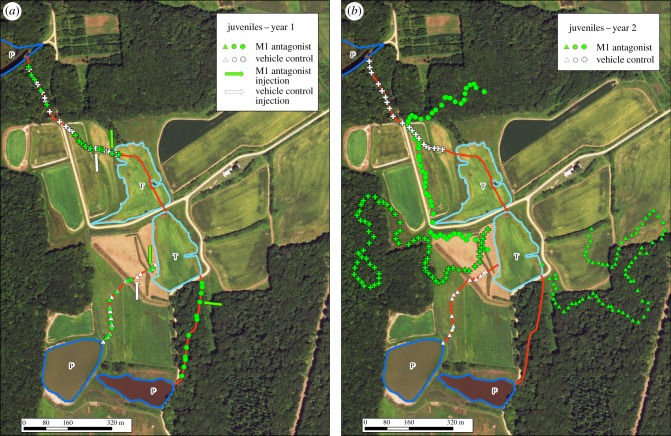
Successful navigation in year 2 is based on M1 ACh-facilitated memory formation in juvenile turtles during year 1. Representative sample of movement of naive juvenile *Chrysemys picta* from temporary (T) to permanent (P) ponds while treated with either an M1 AChR antagonist or DMSO control and subsequent behavioural testing after their critical period. All naive 3-year-old juveniles (year 1; *a*) in both the control (*n* = 3, white) and the M1 AChR antagonist treatments (*n* = 6, green) followed traditional, predicted paths (red) exactly within traditional error (±3.5 m). When retested the following year (year 2; *b*) as 4 year olds and outside of the critical period, the turtles that had received the M1 antagonist (*n* = 6, green) were unable to navigate successfully. The control animals treated only with the DMSO the year before (*n* = 3, white) navigated paths with typical high precision despite being past the critical period (*b*). Arrows represent the location of injection. The symbol colour/type represents the same individuals between years. Each line of points represents the movement of one individual. (Online version in colour.)

Neither the M1 antagonist nor the DMSO vehicle altered the navigation abilities of naive juveniles. Navigational precision pre- and post-injection did not differ in either group (mean overlap with the 3.5 m buffer in both juvenile groups was not significantly different from the historical comparison; *p* > 0.999; figures 2 and 3*a*). All naive juvenile turtles were successful in navigating to alternative aquatic habitat (figure 3*a*), and this level of precision was consistent with that observed previously in this system [20,28]. These results indicate that the M1 AChR antagonist did not influence any factors driving successful navigation beyond spatial memory.

### (c) Turtle navigation is spatial, cognitive, and contingent upon a critical period

After the naive juveniles successfully navigated the historical paths and found permanent water sources (July 2016), we returned them to the donor population. The following year (June 2017), we recaptured all individuals that had aged out of the critical period (i.e. that were now 4 yo; DMSO controls, *n* = 2, one female, one male; M1 antagonist, *n* = 6, three female, three male) and reexamined their ability to navigate these very same paths. In our system, turtles that do not receive navigational experience prior to age 4 (the close of the critical period) are unable to navigate or learn paths [20]. Thus, if our juveniles formed memories of the paths the first year of the experiment (2016), they should be able to navigate the paths in the second year (2017). However, if they had been prevented from forming memories of the paths the previous year (i.e. if memory formation was blocked by the M1 AChR antagonist in 2016), they should be unable to navigate the paths the second year (2017) as they are outside of the critical period.

We found that navigation of complex habitat is dependent upon early spatial cognitive experiences. Turtles that received the DMSO control as 3 yo (2016) selected and successfully navigated the same path again as 4 yo (2017) and did so with the expected traditional precision (figures 2 and 3*b*). These control animals maintained the ability to form memories during their initial journey (2016) as 3 yo and were able to recall those memories during the following year (2017) as 4 yo. In stark contrast, turtles that received the M1 antagonist as 3 yo (2016) and had spatial memory formation blocked were unable to navigate any route to alternative water at 4 yo (2017; figures 2*b* and 3*b*), despite successful navigation the previous year (figures 2*b* and 3*a*). Even though the experimental group was able to navigate successfully as 3 yo, because of our treatment with the M1 antagonist, they were unable to form memories of that experience. Consequently, when those animals were forced to navigate the same paths the following year at 4 yo (after their critical period), they were unable to do so.

Our results suggest that in order to navigate successfully as adults, juveniles need cognitive experiences (in this case, spatially explicit memories associated with the M1 subtype of AChR) prior to the end of their critical period. The more simple, non-cognitive navigation strategy used by juveniles of following local cues on a route [28] was not sufficient for long-term success without cognition. Without the ability to convert spatial experiences into memories, individuals were unable to navigate after the critical period. These results highlight the importance of understanding the developmental patterns of a species during the study of its cognition and greatly enhance our understanding of the evolution of advanced cognition from neurophysiological and developmental perspectives.

## 3. Conclusion

Our data suggest that adult *C. picta* use spatial memory to navigate complex habitats and that these memories are formed within a critical learning period ending at age 4. When treated with an M1 antagonist, experienced adults immediately deviated from their navigation paths while naive juveniles showed no change in navigation ability. This demonstrates that the antagonist only disrupts the memory aspects of navigation and not other aspects of the navigation process itself (e.g. sensory input, motor function, motivation).

By testing the same juveniles both before and after their critical period, we demonstrated the importance of cognitive experience in successful navigation, and that these experiences are facilitated by M1 AChRs. Despite successfully navigating with high precision within the critical period, naive juveniles were prevented from forming memories of their navigation experience when under the influence of the M1 antagonist. Thus, when retested a year later (outside of the critical period), the M1 antagonist-treated turtles were unable to navigate successfully. By contrast, the juveniles treated with the DMSO control were able to form new memories of their migratory routes. When retested the following year outside of the critical period, these juveniles were able to recall the spatial memories formed during a single previous spatial experience and thus navigated paths with high precision 1 year later.

Our study provides strong evidence that the neural mechanism regulating navigation in this system is cholinergic in nature. Specifically, navigation behaviours seem to be facilitated by the M1 AChR type and are predicated on the cognitive processing of spatial memories. To our knowledge, this is the first evidence to suggest a specific neuro-molecular mechanism of complex navigation in a free-ranging wild animal. Moreover, our work is the first to experimentally demonstrate the neurological basis of the cognitive processes involved in the refinement of navigation ability through the formation and subsequent recall of spatial memories during migration. These two advances were possible in part due to our ability to integrate behavioural neuroscience and behavioural ecology in a natural landscape. Laboratory-based studies are critical to our fundamental understanding of the neurological mechanisms underlying spatially explicit behaviours, but many are potentially limited in their application to behavioural decision-making in natural systems on account of their small spatial scale, simplified environment, and often simplified behavioural tasks [31]. By manipulating free-living animals as they perform natural behaviours in the wild, field-based behavioural neuroscience is able to examine the role of neurochemical mechanisms in regulating cognitive processes in an animal as it performs natural behaviours and experiences the related suite of natural stressors (*sensu* [31]). Thus, such studies provide a more robust understanding of the neural and cognitive mechanisms behind the complex, ecologically relevant behaviours necessary to learn migration patterns. We encourage future research in behavioural neuroscience to integrate ecologically relevant questions and field-based approaches, not only for a more robust understanding of how neural mechanisms control behaviour, but also to enhance future translational and applied work [31].

The support for the role of M1 AChR in spatial memory acquisition and recall during navigation in a basal species provides the impetus for examining the mechanistic underpinnings of spatial cognition and navigation in other taxa [31]. Indeed, the field is currently experiencing a renaissance in the approach to studying cognition and its neurological mechanisms across vertebrates (e.g. [32]). Even considering the possibility that mammalian- or avian-like processes (e.g. critical periods in learning) and neurological mechanisms (e.g. role of ACh in cognition) are fundamental to the formation and recall of memories in a reptile greatly expands our understanding of the evolution of animal cognition (see also [31,33]). This expanded phylogenetic perspective provides a contextual framework for the future study of individual variation in learning, ontogenetic patterns in cognitive function (e.g. age-related memory loss), and the neuro-physiological and morphological correlates of the critical learning period itself.

## 4. Material and methods

### (a) Model species

The present study focused on *C. picta*, a long-lived (approx. 25 yr) pond turtle inhabiting a variety of fresh water bodies across the northeastern USA [34]. This species has been studied extensively, particularly in reference to movements, orientation and navigation (e.g. [27,30,35–37]), learning (e.g. [16,20,38]), and spatial memory (e.g. [17,28]).

### (b) Study system

We conducted our study at Chesapeake Farms, a 3300 acre wildlife management and agriculture research area in Kent Co., MD, USA (39.194° N, 76.187° W; [20]). The focal portion of the site is composed of five wetland impoundments (three permanent and two temporary). The temporary ponds (each approx. 2.5 ha in area) have experienced a rapid draining (the entire pond is drained in approx. 24 h) each summer for nearly 100 years for the purpose of wetland management. After draining, resident turtles leave the pond and navigate to alternative aquatic habitats using one of four very precise (±3.5 m), complex, and highly predictable routes (animals use the same routes year after year) [20,28,29]. Naive turtles must navigate this site prior to age 4 to be able to successfully navigate as adults [20,28,29].

The extreme precision in navigation, the repeatability of complex movements, and the critical period for learning to navigate seen in this system provide a unique opportunity to make very specific predictions about the nature of navigation of these animals. We used this unique system to test the importance of M1 AChR for the process of navigation in free-living animals. Unlike some other model systems (e.g. migrating birds or sea turtles), our system allows us to monitor animal navigation in real-time with exceptionally high spatial and temporal resolution. The historical paths provide us with very precise predictions about the turtles' movement and allow us to easily document when navigation abilities are lost. Thus, we can clearly document the behavioural ramifications of experimental manipulation of cognition in freely navigating animals.

### (c) Assessing turtle movements during migration

We used radiotelemetry to investigate the means by which turtles navigate to alternative aquatic sites after the draining as per our previous work [20,27–29]. We captured turtles using baited hoop traps and basking traps, and fitted them with radiotransmitters (models RI-2B or BD-2, Holohil Systems, Ltd, Ontario, Canada, less than 5% body mass). We tracked turtles *via* remote triangulation, taking high precision (spatial resolution of 2.5 m) locations on all animals for the entirety of their terrestrial journey [29].

### (d) Pharmacological manipulation and assessing the role of cognitive experiences

We performed pharmacological manipulations of spatial memory in turtles in July 2016. We allowed individuals to navigate approximately 1/4–1/2 their routes naturally prior to manipulation. Adults (*n* = 7) were then given VU0255035 (*N*-(3-oxo-3-(4-(pyridine-4-yl)piperazin-1-yl)propyl)benzo[c][1,2,5]thiadiazole-4-sulfonamide hydrate; 1.3 mg kg^−1^ in 2% v/v DMSO/saline; Sigma-Aldrich), a specific M1 AChR antagonist [22,24]. Dosage was determined by extrapolating the smallest effective dose for the drug in the rodent literature (0.1 mg kg^−1^) [22] to turtles (1.3 mg kg^−1^) based on the proportional difference between the typical minimum dose of scopolamine in rats and from our own work in turtles (13×, as per refs [16,17,20]). After swabbing the turtle's caudal peritoneal sinus with 70% isopropanol, injections were given interperitoneally with a 22 ga needle. As a control, we also injected additional adults (*n* = 3) with a DMSO vehicle (2% v/v with saline) at identical dosage volumes based on body mass. All injectables were filtered through a 0.22 µm filter prior to delivery. All handling and injections were done in the field at the point of capture and all were completed in less than 30 s. See Roth *et al*. [29] for a full description and demonstration of field and laboratory procedures.

To control for non-cognitive aspects of the M1 AChR antagonist on navigation, we trapped naive juvenile turtles in a distinct population (Chester River Field Research Station, Queen Anne's Co., MD; 39.222° N, 76.986° W), and released them at our study site. We pharmacologically manipulated and radiotracked the naive juveniles in the same manner as the adults, allowing them to navigate approximately 1/4–1/2 of a route, before injection with either the M1 AChR antagonist (*n* = 6; all 3 yo; three female, three male) or DMSO as a vehicle control (*n* = 3; one 2 yo, male; two 3 yo, one male, one female). These injections occurred in the same manner as and at the same dosage concentrations used in adults. Approximately one week post-draining, all naive juveniles had navigated from the temporary pond and were subsequently returned to the donor site.

To investigate the role of spatial memory recall and cognitive experiences for successful navigation, the following year (June 2017) we recaptured all individuals that had aged out of the critical period (i.e. that were now 4 yo; DMSO controls, *n* = 2, one female, one male; M1 antagonist, *n* = 6, three female, three male), and again released them in the test site's temporary pond. We radiotracked these individuals again without manipulation and examined their ability to navigate the very same paths.

### (e) Statistical analyses

We compared the navigation patterns among treatment groups as per our previous analyses [20,28,29]. Briefly, we compared path specificity and precision by calculating the spatial variability of paths taken by each individual as the distance of each individual from the traditional route. Using LOAS (Ecological Software Solutions) and ArcGIS 10.2.1 (Esri Industries), we first documented the traditional path taken by resident adults in previous years [27,28]. Given that resident paths are historically accurate to 3.5 m, we statistically compared all turtle movements to this template by calculating the mean distance of each location for each individual (using the individual turtle as sampling unit) from that of the historical paths. We analysed these distances across treatment groups with a general linear model with Fisher's LSD post hoc comparisons (as per [27,28]). We also produced a measure of the deviation of movements relative to the residents' paths by examining the proportion of points that overlapped the traditional 3.5 m path.

## Data accessibility

Data available from the Dryad Digital Repository at: https://doi.org/10.5061/dryad.457tt13 [39].
